# Short-term changes in behavioral determinants following a theory-based workplace musculoskeletal health program among automobile manufacturing workers: a two-cluster program evaluation

**DOI:** 10.3389/fpubh.2026.1888259

**Published:** 2026-07-07

**Authors:** Jung Young Cho, Gaeun Kim

**Affiliations:** 1 Graduate School, Keimyung University, Daegu, Republic of Korea; 2 Yeungnam University Medical Center, Daegu, Republic of Korea; 3 College of Nursing, Keimyung University, Daegu, Republic of Korea

**Keywords:** manufacturing workers, musculoskeletal disorders, occupational health nursing, theory of planned behavior, workplace health promotion

## Abstract

**Background:**

Work-related musculoskeletal disorders (WMSDs) are a major occupational and public health burden among manufacturing workers. Existing workplace interventions often emphasize ergonomic assessment or generic safety education, with limited use of behavior-change theory. We examined short-term changes in Theory of Planned Behavior (TPB)-based determinants and self-reported preventive behavior following an on-site, nurse-delivered musculoskeletal-health program.

**Methods:**

A two-cluster, cluster-allocated controlled program evaluation was conducted in two plants in City D, Republic of Korea (*N* = 69; intervention *n* = 34, control *n* = 35), with assessments at baseline, week 4, and week 8. Male field workers with self-reported musculoskeletal symptoms identified through workplace ergonomic risk assessment were recruited; plant-level allocation was random, although balance cannot be assumed with only two clusters. The 4-week intervention comprised 4 weekly education sessions plus thrice-weekly small-group activities; controls received routine workplace safety education without attention-matching. Outcomes were four TPB constructs, a single-item self-reported behavior item, and NIOSH-based musculoskeletal symptoms. Generalized estimating equations modeled continuous outcomes; dichotomous outcomes were examined using Pearson chi-square and exact tests as appropriate. The study was not prospectively registered.

**Results:**

Significant group-by-time interactions were observed for PBC (χ^2^ = 12.92, *p* < 0.001) and behavioral intention (χ^2^ = 10.88, *p* = 0.001), but not for attitude or subjective norms. Follow-up between-group standardized mean differences were *d* = 0.58 (95% CI 0.10–1.06) for PBC and *d* = 0.40 (95% CI −0.08–0.87) for intention. Self-reported preventive behavior increased in the intervention group from 29.4% at baseline to 100.0% post-intervention and 82.4% at follow-up, compared with 31.4% and 25.7% in controls at the corresponding timepoints (both between-group *p* < 0.001; follow-up RR 3.20, 95% CI 1.79–5.74); the 100% post-intervention rate reflects single-item self-report limitations. Overall NIOSH symptom-carrier status did not differ between groups at any timepoint.

**Conclusion:**

The program was accompanied by short-term changes in PBC, behavioral intention, and self-reported preventive behavior. Given the two-cluster design, non-attention-matched control, single-item behavioral outcome, and absence of prospective registration, these findings should be interpreted as feasibility- and mechanism-oriented signals rather than evidence of efficacy.

## 1 Introduction

Work-related musculoskeletal disorders (WMSDs) are conditions caused by repetitive motions, awkward postures, and sustained physical loads during occupational activities. Globally, musculoskeletal disorders affect over 1.71 billion people and represent the leading contributor to years lived with disability (1). WMSDs are not solely a clinical or ergonomic problem; they constitute a public health issue because they reduce work ability, increase sickness absence, impair quality of life, and generate substantial social and economic costs. Automobile manufacturing workers are a high-risk occupational group, with a recent systematic review and meta-analysis reporting an elevated WMSD prevalence in this sector (2). In the Republic of Korea, WMSDs account for 65.1% of all recognized occupational diseases (3), and Korean industrial surveillance similarly documents elevated WMSD risk across manufacturing sectors (5, 6).

Workplace WMSD prevention strategies have predominantly focused on ergonomic risk assessment and engineering controls, complemented by generic safety education (4). However, despite a regulatory framework mandating ergonomic hazard assessments in Korean industry (4), WMSD prevalence has remained elevated, suggesting that environmental and engineering interventions alone are insufficient. Systematic reviews consistently indicate that the addition of individual-level behavioral components to workplace prevention strategies enhances their effectiveness (7–9). However, evidence remains limited regarding theory-informed workplace programs targeting WMSD-related preventive behaviors among manufacturing workers exposed to high physical loads, and few studies in Asian industrial settings have tested behavioral mechanisms such as perceived behavioral control and behavioral intention.

The Theory of Planned Behavior (TPB) (10) posits that behavioral intention—shaped by attitude, subjective norms, and perceived behavioral control (PBC)—is the most proximal determinant of behavior. Meta-analytic evidence has confirmed the TPB's predictive validity across diverse health domains (11, 12), with PBC consistently emerging as a strong predictor of sustained behavior change in occupational health settings (13). A recent randomized trial demonstrated the efficacy of a TPB-based health education program for ergonomic posture among hospital computer users (14); evidence remains limited, however, for TPB-informed workplace programs in manufacturing populations exposed to high physical loads. Theory-driven occupational health interventions generally produce larger and more sustained effects than atheoretical approaches (7, 15).

From an implementation perspective, on-site occupational health nurses are well positioned to operationalize theory-driven workplace health promotion because they bridge worker-, supervisor-, and organization-level health systems. Occupational health nursing services are embedded within Korean industrial sites under the Occupational Safety and Health Act (4), yet their potential role as an implementation workforce for theory-based behavioral interventions—rather than only as providers of routine health surveillance—has rarely been evaluated empirically.

Against this background, this study aimed to develop a TPB-based workplace health promotion program—coordinated and delivered by on-site occupational health nursing staff—for musculoskeletal health among automobile manufacturing workers, and to examine short-term changes following its implementation in: (i) behavioral determinants (attitude, subjective norms, PBC, behavioral intention); (ii) self-reported health behavior practice; and (iii) self-reported musculoskeletal symptoms.

## 2 Materials and methods

### 2.1 Study design and framing

This study was conducted as a two-site, cluster-allocated controlled workplace program evaluation with pre-, post-, and short-term follow-up assessments, rather than as a fully powered multi-cluster randomized trial. Two automobile manufacturing plants of comparable size (approximately 200 workers each) in City D, Republic of Korea, were selected. Plant-level allocation was chosen to prevent cross-group contamination, because individual randomization within a single plant would risk intervention diffusion through shared work environments, break rooms, and peer interactions.

Because only two clusters were available, conventional cluster-randomized-trial inference standards cannot be met (21), and the findings are interpreted as preliminary program evaluation evidence. The study is reported with reference to the CONSORT extension for cluster randomized trials (22), while acknowledging that a two-cluster design does not satisfy that standard. GEE with an exchangeable working correlation structure was used to model the repeated continuous TPB outcomes at the individual level; this modeling approach was used to describe repeated-measure trajectories transparently and not to claim cluster-randomized-trial inference.

### 2.2 Setting, participants, and recruitment

Both plants operated comparable assembly-line manufacturing processes with similar physical task profiles. Following the plant-level workplace ergonomic risk assessment, male field workers who had reported musculoskeletal symptoms in any body region within the past 12 months were invited to participate. The two plants were allocated to intervention or control at the cluster level by an independent third party who drew, from an opaque box, one of two cards each bearing a plant name; the plant drawn first was assigned to the intervention condition and the second to the control condition. Because allocation was applied to two pre-existing plants rather than to individuals, individual-level allocation concealment was not applicable; workers and on-site staff were necessarily aware of their plant's assignment once the program began. The two plants were comparable before the intervention in workforce size (approximately 200 workers each), assembly-line manufacturing processes, physical task profiles, and the schedule of mandatory occupational safety education; measured worker-level baseline characteristics are reported in Table 1. However, because only two clusters were available, this procedure should not be interpreted as achieving randomization-based balance, and the findings are interpreted accordingly.

**Table 1:** Baseline characteristics of participants (*N* = 69).

Characteristic	Intervention (*n* = 34)	Control (*n* = 35)	*t*/χ^2^	*p*
Age ≥ 40 years, *n* (%)	12 (35.3)	16 (45.7)	0.78	0.465
BMI ≥ 25 kg/m^2^, *n* (%)	4 (11.8)	8 (22.9)	1.48	0.342
Self-rated health ‘good' or better, *n* (%)	7 (20.6)	9 (25.7)	0.25	0.777
Workload rated ‘heavy', *n* (%)	23 (67.6)	24 (68.6)	0.01	0.997
Regular physical activity, *n* (%)	8 (23.5)	11 (31.4)	0.54	0.592
Job tenure (years), M ± SD	10.9 ± 5.2	10.8 ± 4.3	0.10	0.923
NIOSH symptom carrier, *n* (%)	25 (73.5)	20 (57.1)	2.04	0.153

BMI, body mass index; M, mean; SD, standard deviation. Regular physical activity defined as engaging in moderate-to-vigorous physical activity at least three times per week. NIOSH symptom carrier defined as reporting symptoms with frequency of at least monthly or duration of at least 1 week. No statistically significant between-group differences were detected for the characteristics shown; however, with n = 69 this should not be read as evidence of equivalence. Standardized differences, which do not depend on sample size, indicated non-negligible baseline imbalance for the NIOSH symptom-carrier proportion (Cohen's h = 0.35) and for baseline attitude (Cohen's d = –0.55).

Inclusion criteria were: (a) employment as a field worker; (b) self-reported musculoskeletal symptoms in at least one body region (e.g., shoulder, back, knee) related to work, identified through the workplace ergonomic risk assessment within the past 12 months. Exclusion criteria applied to workers unable to participate during the scheduled intervention period for medical or other reasons. Sample size was calculated using G^*^Power 3.1.7 (16) (independent-samples *t*-test; α = 0.05, *d* = 0.8, power = 0.80), requiring 26 workers per group. Accounting for an anticipated 30% attrition rate based on prior workplace intervention research, 37 workers per cluster were recruited (*N* = 74). During the intervention period, three workers from the intervention group and two from the control group withdrew due to health-related absence, business travel, or resignation (overall attrition 9.32%), yielding a final analyzed sample of 69 (intervention plant: *n* = 34; control plant: *n* = 35).

Participation was voluntary, and workers were informed of the study's purpose, procedures, expected benefits and potential risks, and the possibility of allocation to either the intervention or control condition. Workers were also informed that they could withdraw from the study at any time. Data were managed anonymously and used solely for the stated research purposes. Workers were aware of the group to which their plant was allocated; outcome assessors were not blinded, as is standard in pragmatic cluster-allocated workplace evaluations. Recruitment and consent procedures were conducted separately from supervisors and plant management, and workers were informed that participation or non-participation would not affect employment status, workplace evaluation, or access to occupational health services.

### 2.3 Ethical considerations and trial registration

The study was conducted in accordance with the Declaration of Helsinki and approved by the Institutional Review Board of the coordinating university (IRB No. 40525-202410-HR-062-03). All participants provided written informed consent prior to baseline assessment. The study was not prospectively registered prior to enrolment; at the time of implementation, it was conducted as an institutionally approved workplace health promotion program evaluation. Because the study involved prospective allocation of workers to a health-related behavioral intervention with structured outcome assessment, the absence of prospective registration is acknowledged as a transparency limitation, and the manuscript is presented as preliminary program evaluation evidence rather than as a definitive efficacy trial. The study has not been registered retrospectively, and this is openly reported as a study limitation.

### 2.4 Intervention

The intervention is described in summary form in Table 2 [TIDieR framework (23)] and session-by-session in Table 3. Briefly, the 4-week intervention was grounded in the TPB and targeted the cognitive-motivational determinants of preventive musculoskeletal health behavior. 4 weekly 60-min group education sessions were combined with thrice-weekly 10–15-min small-group activities, including guided stretching, peer experience sharing, and case-based problem-solving. Behavior-change techniques included instruction, demonstration, behavioral rehearsal, goal-setting, and self-monitoring through pledges. Sessions were planned and delivered by on-site occupational health nursing staff (registered nurses with occupational health experience), who also coordinated peer ‘Workplace Health Guardian' leaders supporting between-session activities. The nursing staff were responsible for the full operational chain—needs assessment, session delivery, between-session support, fidelity recording, and outcome assessment—linking individual worker behavior change to the plant's routine occupational health services.

**Table 2:** Intervention description according to the TIDieR framework.

TIDieR item	Description
1. Brief name	TPB-based workplace health promotion program for musculoskeletal health, delivered by on-site occupational health nursing staff
2. Why (rationale, theory)	Grounded in the Theory of Planned Behavior; targets attitude, subjective norms, perceived behavioral control, and behavioral intention as proximal determinants of preventive behavior
3. What (materials)	Education slides, case-study handouts, ergonomic posture and stretching guides, goal-setting and pledge forms, posters, videos, session delivery checklists
4. What (procedures)	Group education combined with thrice-weekly small-group activities (guided stretching, peer experience sharing, case-based problem-solving, role-play); behavior-change techniques included instruction, demonstration, behavioral rehearsal, goal-setting, and self-monitoring
5. Who provided	On-site occupational health nursing staff (registered nurses with occupational health experience) planned and delivered all sessions, supervised peer ‘Workplace Health Guardian' leaders, and recorded fidelity data
6. How (mode of delivery)	Face-to-face group sessions; in-person small-group activities
7. Where (setting)	Worker health room and adjacent meeting space within the intervention plant
8. When and how much	Four weekly 60-minute group sessions plus thrice-weekly 10–15-minute small-group activities over a 4-week period
9. Tailoring	Case-based discussions referenced workers' specific job tasks and reported symptom regions; sessions incorporated findings from the workplace ergonomic risk assessment
10. Modifications	No protocol modifications during implementation
11. How well (fidelity, planned)	Session attendance and nurse-completed delivery checklists
12. How well (fidelity, actual)	Attendance and checklist completion reported descriptively; no independent fidelity audit was conducted
Control condition	Standard occupational safety education at the control plant (generic briefings covering workplace hazards, personal protective equipment use, and emergency procedures); no TPB-based behavioral content; no additional study-related contact beyond outcome assessment. Intervention-group workers also continued to receive these briefings on the same schedule

TIDieR, template for intervention description and replication; TPB, theory of planned behavior.

**Table 3:** Session-by-session overview of the TPB-based workplace health promotion program.

Session	Topic	Key activities	TPB	Duration
1	Musculoskeletal health knowledge and motivation	Health-consequence education; team formation; pledge signing; goal setting	A, S, P, I	60 min
2	Skills building and social support	Coping strategies; supervisor notification; role-play exercises	A, S, P, I	60 min
3	Practice consolidation	Case sharing; ergonomic assessment feedback; guided stretching	A, S, P	60 min
4	Maintenance and self-management	Ergonomic principles review; improvement pledges; barrier discussion	A, P, I	60 min

A, attitude; S, subjective norms; P, perceived ^behavioral^ control; I, ^behavioral^ intention. All sessions were planned and facilitated by on-site occupational health nursing staff. Sessions were complemented by thrice-weekly 10–15-min small-group activities throughout the 4-week intervention period.

Fidelity was monitored against the planned protocol using session attendance registers, nurse-completed delivery checklists for each of the four group sessions and the thrice-weekly small-group activities, and a brief structured orientation for the peer ‘Workplace Health Guardian' leaders covering the program schedule, the stretching demonstration, and the peer-support role. Following the TIDieR framework (23) and the treatment-fidelity recommendations of the NIH Behavior Change Consortium (24), dose delivered and dose received are reported descriptively. All four weekly group sessions and the thrice-weekly small-group activities were delivered on-site as planned by the occupational health nursing staff, with no protocol modifications during implementation; of the 37 intervention-group workers enrolled, 34 (91.9%) attended the full program and completed all assessments, while 3 discontinued because of scheduling conflicts, business travel, or resignation. No independent fidelity audit was conducted; fidelity of receipt and enactment could not be objectively verified, and these descriptive figures should be interpreted accordingly.

#### 2.4.1 Control condition

Workers in the control plant received the plant's standard occupational safety education as scheduled. This comprised generic safety briefings covering workplace hazards, personal protective equipment use, and emergency procedures, without TPB-based behavioral content. Intervention-group workers also continued to receive these mandatory safety briefings on the same schedule as control workers; this contact was identical in both groups. Beyond the scheduled briefings and outcome-assessment sessions, no additional study-related contact occurred with control-plant workers during the intervention period.

#### 2.4.2 Attention asymmetry

Because the intervention group received substantial additional contact time, peer engagement, and structured small-group activities that the control group did not, any observed changes may partly reflect differential attention, contact, or peer-support exposure rather than TPB-specific behavior-change mechanisms. This attention asymmetry is discussed in Section 4.5.

### 2.5 Outcome measures

Outcomes were structured around the TPB framework. Perceived behavioral control and behavioral intention—as the most proximal TPB determinants targeted by the program—are reported as the primary outcomes of interest. Attitude, subjective norms, self-reported health behavior practice, and self-reported musculoskeletal symptom prevalence are reported as additional outcomes. Because the study was not prospectively registered, the distinction between primary and additional outcomes should be regarded as analytic structure rather than as a registered hypothesis hierarchy.

TPB constructs were assessed using 4-item Likert (5-point) scales adapted from the direct-measurement instruments developed by Terry and O'Leary (17) and previously translated and used in Korean populations. The original instrument reliability was Cronbach's α = 0.81 (attitude), 0.73 (subjective norms), 0.82 (PBC), and 0.85 (intention); in the present sample, Cronbach's α was 0.83 for all four constructs. Health behavior practice was assessed at each timepoint using a single dichotomous self-report item that asked whether, during the preceding 4 weeks, the worker had practiced the target behaviors (correct working posture and stretching), with response options 'yes (practiced)' or 'no (did not practice)'. A single pragmatic item was used because the evaluation was embedded within routine occupational health services with minimal additional response burden, and because no validated short adherence scale for these specific workplace behaviors was available in Korean at the time of the study; the item was reviewed for clarity by the on-site occupational health nursing staff before use but was not formally piloted or psychometrically validated. The item indexes only whether the behavior was reportedly performed and does not capture its frequency, duration, or quality, and does not constitute objectively verified adherence. Because outcome assessors were not blinded and intervention-group workers were aware of the program's behavioral goals, this item is particularly susceptible to social-desirability bias and demand characteristics, most acutely at the immediate post-intervention assessment; its results are therefore interpreted only as a self-reported behavioral-adoption signal. Musculoskeletal symptoms were assessed using the Korean Occupational Safety and Health Agency symptom survey (KOSHA, 2018) (18), an instrument grounded in the Nordic standardized questionnaire format (19) and NIOSH surveillance criteria (20), covering six body regions (neck, shoulder, arm, hand, back, leg) with self-reported duration (five categories), intensity (four categories), and frequency (five categories). Workers were classified as ‘symptom carriers' per NIOSH surveillance criteria when symptom frequency was at least monthly or duration was at least 1 week (20).

### 2.6 Data collection

Data were collected between February and March 2025 at baseline (T0), post-intervention (T1; 4 weeks), and follow-up (T2; 8 weeks from baseline). Self-report questionnaires were distributed and collected on-site under standardized conditions; outcome assessment was not blinded owing to the cluster-allocated program evaluation design.

### 2.7 Statistical analysis

Data were analyzed using IBM SPSS Statistics version 29.0 (IBM Corp., Armonk, NY, USA). Continuous variables are presented as mean ± standard deviation; categorical variables as frequency and percentage. Baseline between-group comparisons used independent-samples *t*-tests or Pearson chi-square tests. Because non-significant baseline tests with *n* = 69 cannot be read as evidence of equivalence, baseline balance was additionally summarized using standardized differences that do not depend on sample size (Cohen's d for continuous variables and Cohen's h for proportions; values ≥ 0.20 were regarded as non-negligible imbalance). Mauchly's test of sphericity was performed and not satisfied; multivariate Pillai's trace was therefore used for exploratory repeated-measures analyses. For the main analysis of the four TPB constructs across baseline, post-intervention, and follow-up, GEE with an exchangeable working correlation structure was applied at the individual level to model group (G), time (T), and group × time (G × T) effects. Significance of effects is reported as the GEE Wald χ^2^ statistic and corresponding *p*-value for descriptive purposes; in addition, between-group standardized mean differences (Cohen's d) with 95% confidence intervals are reported at post-intervention and follow-up so that interpretation rests on the direction and precision of effect estimates rather than on *p*-values alone. The GEE models were used to describe repeated-measure trajectories at the individual level transparently; they should not be interpreted as overcoming the inferential limitations of the two-cluster design. Between-group comparisons of dichotomous outcomes (health behavior practice; symptom-carrier prevalence) used Pearson chi-square tests, with Fisher's exact tests where cell counts were small; within-group change in symptom-carrier status over time was assessed using McNemar's exact test. Between-group effects for dichotomous outcomes are additionally summarized as absolute risk differences and risk ratios with 95% confidence intervals (25). Where one group reached a 0% or 100% response and the risk ratio was therefore undefined or unstable, the risk difference with its confidence interval is reported as the primary effect estimate and the comparison is flagged as statistically extreme. Body-region-specific musculoskeletal symptom analyses (six regions × three subdomains [duration, intensity, frequency] × three timepoints) are presented as uncorrected exploratory analyses; no adjustment for multiple comparisons was applied because these 54 comparisons were hypothesis-generating rather than confirmatory. To make the resulting family-wise error inflation explicit, we note that a Bonferroni-corrected threshold would be approximately p < 0.001, and no single body-region result is interpreted as a stand-alone finding. An additional exploratory analysis among workers showing any symptom improvement is provided in Supplementary File S2. Missing data were minimal and analyzed under a missing-at-random assumption using available-case analysis. Significance was set at *p* < 0.05 for descriptive purposes.

## 3 Results

### 3.1 Participant characteristics

**Figure 1:**
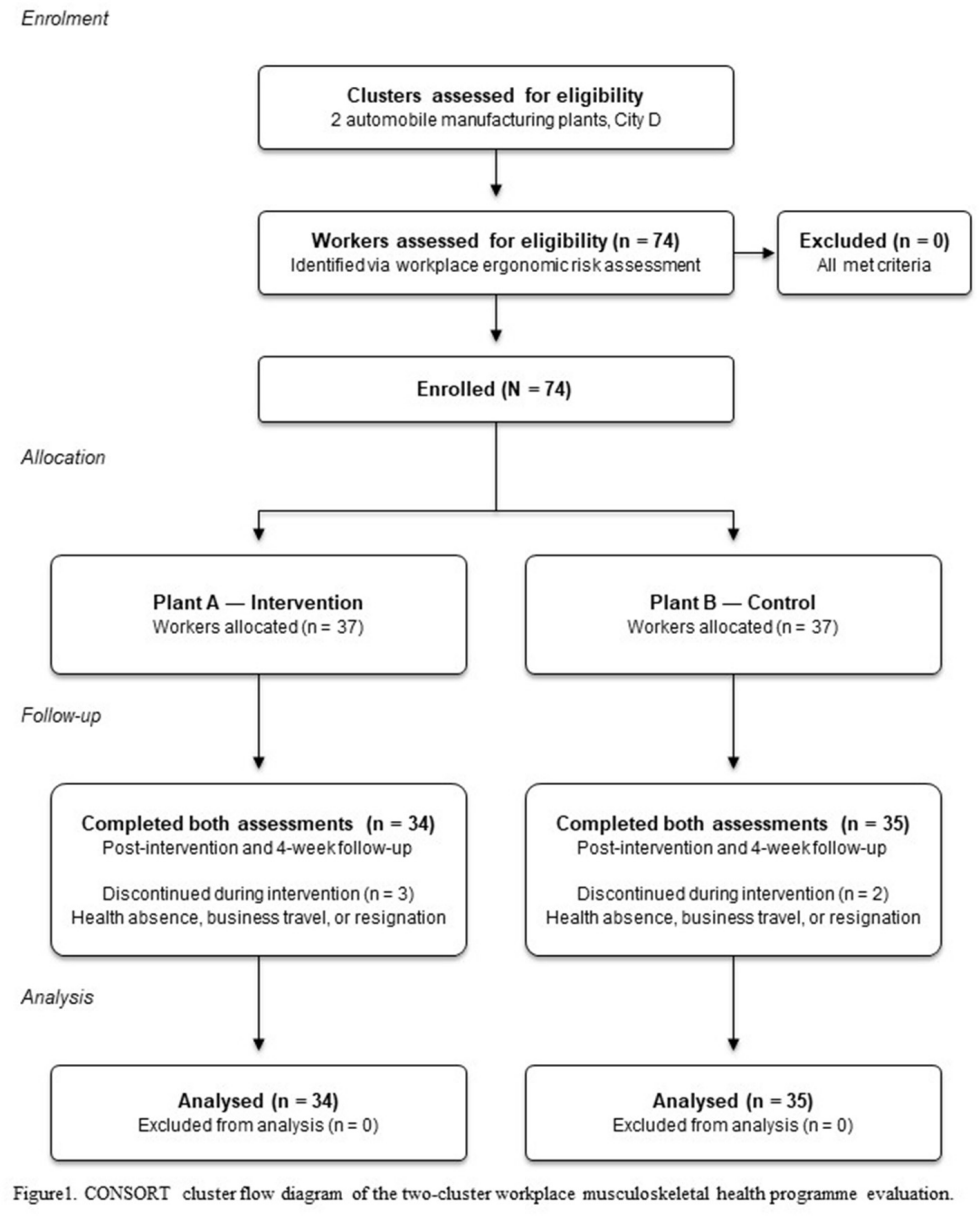
CONSORT cluster flow diagram of the two-cluster workplace musculoskeletal health program evaluation. Two automobile manufacturing plants in City D, Republic of Korea, were allocated at the cluster level to intervention or control. Within each cluster, 37 workers with self-reported musculoskeletal symptoms identified through the workplace ergonomic risk assessment were enrolled (*N* = 74). Three workers in the intervention group and two in the control group withdrew during the intervention period (attrition 9.32%); 34 intervention and 35 control workers were included in the final analysis at post-intervention (week 4) and follow-up (week 8).

Of 74 enrolled workers, 69 completed all assessments (overall attrition 9.32%; Figure 1). All participants were male field workers in automobile manufacturing. Workers aged 40 years or older accounted for 35.3% of the intervention group and 45.7% of the control group (χ^2^ = 0.78, p = 0.465). Mean job tenure was 10.9 ± 5.2 years in the intervention group and 10.8 ± 4.3 years in the control group (*t* = 0.10, *p* = 0.923). BMI ≥ 25 kg/m^2^ was reported by 11.8% of intervention and 22.9% of control workers (χ^2^ = 1.48, *p* = 0.342). Workers rating their health as ‘good' or better made up 20.6% of the intervention and 25.7% of the control group (χ^2^ = 0.25, *p* = 0.777). Self-reported workload was rated as ‘heavy' by 67.6% (intervention) and 68.6% (control) (χ^2^ = 0.01, *p* = 0.997). Regular physical activity was reported by 23.5% (intervention) and 31.4% (control) (χ^2^ = 0.54, *p* = 0.592). At baseline, 73.5% of intervention and 57.1% of control workers met the NIOSH symptom-carrier criterion (χ^2^ = 2.04, *p* = 0.153) (Table 1). Although no characteristic reached statistical significance at the α = 0.05 threshold, *p* > 0.05 with *n* = 69 cannot be interpreted as evidence of equivalence; clinically meaningful residual imbalance—particularly the higher baseline NIOSH symptom-carrier rate in the intervention group (73.5 vs. 57.1%; standardized difference Cohen's *h* = 0.35, a non-negligible imbalance)—cannot be excluded. The implication of this baseline asymmetry, including the possibility of regression to the mean in the more-symptomatic group, is addressed in Section 4.5.

At baseline, all four TPB constructs were comparable between groups except for attitude, which was lower in the intervention group than in the control group (2.90 ± 0.76 vs. 3.33 ± 0.80; *t* = −2.31, *p* = 0.024; standardized mean difference *d* = −0.55, 95% CI −1.03 −0.07). Subjective norms, PBC, and behavioral intention did not differ significantly at baseline (Table 4). Body-region symptom prevalence at baseline was highest for the hand (46.4%), followed by shoulder (37.7%), neck (36.2%), leg (26.0%), back (21.7%), and arm (21.7%); some workers reported symptoms in multiple regions.

**Table 4:** Changes in TPB behavioral determinants by group and time, and GEE model results (*N* = 69).

Variable/group	Baseline	Post	Follow-up	GEE source	χ^2^	*p*
Attitude	–	–	–	G	3.78	0.052
Intervention	2.90 ± 0.76	3.38 ± 1.02	3.18 ± 0.85	T	11.93	0.003
Control	3.33 ± 0.80	3.49 ± 0.90	3.49 ± 0.89	G × T	0.49	0.485
Subjective norms	–	–	–	G	2.63	0.105
Intervention	3.14 ± 0.77	3.28 ± 1.05	3.12 ± 0.91	T	7.98	0.019
Control	2.84 ± 1.05	2.81 ± 1.04	2.74 ± 0.98	G × T	0.42	0.517
PBC	–	–	–	G	2.08	0.150
Intervention	3.00 ± 0.76	3.53 ± 0.84	3.42 ± 0.73	T	21.55	< 0.001^*^
Control	2.94 ± 1.12	2.96 ± 1.18	2.88 ± 1.10	G × T	12.92	< 0.001^*^
Behavioral intention				G	0.35	0.555
Intervention	3.07 ± 0.88	3.54 ± 1.14	3.54 ± 1.14	T	8.70	0.013
Control	3.26 ± 0.61	3.20 ± 0.78	3.16 ± 0.73	G × T	10.88	0.001^*^

PBC, perceived ^behavioral^ control; GEE, ^generalized^ estimating equations; G, group main effect; T, time main effect; G × T, group-by-time interaction. Values are mean ± SD. Cronbach's α in the present sample = 0.83 for all four constructs. A statistically significant baseline between-group difference was observed for attitude (t = −2.31, p = 0.024); subjective norms, PBC, and ^behavioral^ intention did not differ statistically at baseline, although with n = 69 the absence of a significant difference cannot be interpreted as evidence of equivalence. ^*^p < 0.05. Between-group ^standardized^ mean differences (Cohen's d) with 95% CIs at follow-up were: PBC d = 0.58 (0.10–1.06); ^behavioral^ intention d = 0.40 (−0.08–0.87); attitude d = −0.36 (−0.83–0.12); subjective norms d = 0.40 (−0.08–0.88); only the PBC interval excluded zero. GEE models were fitted at the individual level and should not be interpreted as overcoming the inferential limitations of the two-cluster design. An additional exploratory analysis restricted to workers showing any symptom improvement (n = 32) is provided in Supplementary File S2.

### 3.2 Behavioral determinants (TPB constructs)

For PBC, the GEE model identified a statistically significant group-by-time interaction (χ^2^ = 12.92, *p* < 0.001), indicating divergent trajectories across the intervention and control groups at the individual level (Table 4). The intervention group's PBC score moved from 3.00 ± 0.76 at baseline to 3.53 ± 0.84 at post-intervention and 3.42 ± 0.73 at follow-up; the control group's score remained essentially unchanged (2.94 ± 1.12 → 2.96 ± 1.18 → 2.88 ± 1.10). The corresponding between-group standardized mean difference was moderate, with a 95% confidence interval excluding zero (post-intervention *d* = 0.56, 95% CI 0.07–1.04; follow-up *d* = 0.58, 95% CI 0.10–1.06).

For behavioral intention, the GEE model also identified a statistically significant group-by-time interaction (χ^2^ = 10.88, *p* = 0.001) (Table 4). The intervention group's score moved from 3.07 ± 0.88 at baseline to 3.54 ± 1.14 at both post-intervention and follow-up; the control group's score declined slightly from baseline (3.26 ± 0.61) to follow-up (3.16 ± 0.73). The between-group standardized mean difference was small-to-moderate, with confidence intervals that included zero (post-intervention *d* = 0.35, 95% CI −0.13–0.82; follow-up *d* = 0.40, 95% CI −0.08–0.87), indicating an imprecise estimate.

For attitude and subjective norms, no statistically significant group-by-time interactions were detected (attitude χ^2^ = 0.49, *p* = 0.485; subjective norms χ^2^ = 0.42, *p* = 0.517). Both constructs showed modest within-group changes across time, with main effects for time but not for group × time (Table 4).

### 3.3 Self-reported health behavior practice

Baseline self-reported practice rates were comparable between groups (intervention: 29.4%; control: 25.7%; χ^2^ = 0.12, *p* = 0.731; risk ratio [RR] 1.14, 95% CI 0.53–2.46). At post-intervention, all intervention-group workers reported practising the target behavior (100%), vs. 31.4% of the control group (χ^2^ = 35.75, *p* < 0.001); because the intervention-group proportion was 100%, the risk ratio is unstable and the absolute risk difference is reported as the primary effect estimate (+68.6 percentage points, 95% CI +53.2 to +84.0), with Fisher's exact test corroborating the comparison (*p* < 0.001). At the 4-week follow-up, the self-reported practice rate in the intervention group was 82.4%, compared with 25.7% in the control group (χ^2^ = 22.25, *p* < 0.001; risk difference +56.6 percentage points, 95% CI +37.3 to +76.0; RR 3.20, 95% CI 1.79–5.74) (Table 5). Because no intervention-group workers reported non-practice at post-intervention, the corresponding between-group estimate is statistically extreme and is reported in Table 5 only for transparency. Both estimates rest on a single dichotomous self-report item, are susceptible to social-desirability bias and demand characteristics in the immediate post-intervention context, and do not capture practice frequency, duration, or quality; they should be interpreted as a self-reported behavioral-adoption signal rather than as objectively verified behavior change.

**Table 5:** Self-reported health behavior practice by group and time (*N* = 69).

Time	Intervention *n* (%)	Control *n* (%)	χ^2^	*P*
Baseline	10 (29.4)	9 (25.7)	0.12	0.731
Post-intervention	34 (100.0)	11 (31.4)	35.75	< 0.001^*^
Follow-up	28 (82.4)	9 (25.7)	22.25	< 0.001^*^

^*^p < 0.05. Self-reported practice of proper posture and stretching during the preceding 4 weeks at each timepoint. At post-intervention, no intervention-group workers reported non-practice; the resulting between-group comparison is statistically extreme and is reported for transparency. Both estimates are based on a single dichotomous self-report item susceptible to social-desirability bias and cannot capture frequency, duration, or quality of practiced behavior, Effect estimates with 95% CIs: baseline RR 1.14 (0.53–2.46); post-intervention risk difference +68.6 percentage points (+53.2 to +84.0), with the RR unstable owing to the 100% intervention-group rate; follow-up risk difference +56.6 percentage points (+37.3 to +76.0) and RR 3.20 (1.79–5.74). Fisher's exact tests corroborated the post-intervention comparison, where the intervention-group rate reached 100%.

### 3.4 Self-reported musculoskeletal symptoms

NIOSH symptom-carrier status was 73.5% in the intervention group vs. 57.1% in the control group at baseline (between-group Pearson χ^2^ = 2.04, *p* = 0.153), 61.8 vs. 45.7% at post-intervention (χ^2^ = 1.79, *p* = 0.181), and 67.6 vs. 54.3% at follow-up (χ^2^ = 1.29, *p* = 0.256); no between-group difference was statistically significant (Table 6). Within the intervention group, change in symptom-carrier status over time was also non-significant by McNemar's exact test (post-intervention *p* = 0.125; follow-up *p* = 0.500). For descriptive between-group comparison, the risk ratio (intervention vs. control) was 1.29 (95% CI 0.91–1.83), 1.35 (0.86–2.11), and 1.25 (0.85–1.83) across the three timepoints, all including 1. The intervention-group estimate remained numerically higher at every timepoint, consistent with its higher baseline carrier rate. These clinical symptom outcomes should be interpreted separately from the behavioral-determinant and self-reported-practice outcomes reported above: the program was accompanied by changes in behavioral mediators but not by any reduction in overall musculoskeletal symptom-carrier status, and no symptomatic benefit is claimed. Exploratory body-region-specific analyses (six body regions × three symptom subdomains [duration, intensity, frequency] × three timepoints) were uncorrected for multiple comparisons and produced inconsistent patterns across subdomains and timepoints. These analyses are reported in Supplementary File S2 as descriptive signals only and should not be interpreted as evidence of symptom benefit.

**Table 6:** Musculoskeletal symptom outcomes by group and time (*N* = 69).

Outcome/time	Intervention *n* (%)	Control *n* (%)	χ^2^	*p*
NIOSH symptom carrier				
Baseline	25 (73.5)	20 (57.1)	2.04	0.153
Post-intervention	21 (61.8)	16 (45.7)	1.79	0.181
Follow-up	23 (67.6)	19 (54.3)	1.29	0.256

Between-group comparisons at each timepoint used Pearson chi-square tests. Within-group change in the intervention group was additionally assessed using McNemar's exact test and was not statistically significant at post-intervention (p = 0.125) or follow-up (p = 0.500). Descriptive risk ratios were 1.29 (95% CI 0.91–1.83) at baseline, 1.35 (0.86–2.11) at post-intervention, and 1.25 (0.85–1.83) at follow-up.

### 3.5 Additional exploratory analysis

An additional exploratory analysis examining TPB construct changes within the subgroup of workers who showed any symptom improvement (*n* = 32) is reported in Supplementary File S2. Because this subgroup is defined by post-baseline outcome change, the analysis is vulnerable to selection bias and is presented as exploratory and hypothesis-generating only.

## 4 Discussion

### 4.1 Principal findings

This two-cluster workplace program evaluation reports preliminary, feasibility- and mechanism-oriented signals that the program was accompanied by short-term changes at the individual level in perceived behavioral control, behavioral intention, and self-reported preventive health behavior among automobile manufacturing workers. No changes were detected on attitude or subjective norms. Overall NIOSH symptom-carrier status did not differ significantly between groups at any timepoint; exploratory body-region-specific analyses (Supplementary File S2) were uncorrected for multiplicity, inconsistent across subdomains and timepoints, and should not be interpreted as evidence of symptom benefit. Given the two-cluster design, non-attention-matched control condition, self-reported outcomes, baseline asymmetry in symptom-carrier rate, and absence of prospective registration, none of these findings should be interpreted as evidence of intervention efficacy.

### 4.2 Interpretation through the theory of planned behavior

The pattern of construct-specific changes is broadly coherent with the TPB and consistent with meta-analytic evidence that PBC has direct and moderating associations with health behavior, particularly when individuals face practical barriers (13). The absence of changes in attitude and subjective norms likely reflects features of the Korean industrial context: workers receive mandatory safety education under the Occupational Safety and Health Act (4), potentially creating a ceiling effect on baseline safety attitudes. Subjective-norm change typically requires sustained multi-level organizational engagement extending beyond an individually focused 4-week program. The divergent pattern of change across TPB constructs—movement in PBC and intention but not in attitude or subjective norms—is not fully consistent with a simple global plant-level explanation, although unmeasured plant-level confounding and non-specific attention effects cannot be excluded as alternative explanations.

### 4.3 Public and occupational health implications

From a public health perspective, the findings are consistent with the broader argument that workplace health promotion for WMSDs may be relevant beyond information provision alone, with behavioral capability building, peer-supported social learning, and environmental facilitation as candidate components. Theory-driven interventions that target proximal cognitive-motivational determinants alongside practical skill development offer one candidate model for occupational public health programming in high-risk industrial populations, though confirmatory evidence is required. The exploratory body-region-specific symptom analyses (Supplementary File S2) were uncorrected for multiplicity and inconsistent across subdomains and timepoints; in combination with the null finding for overall NIOSH symptom-carrier status, they do not support a conclusion that the program reduced musculoskeletal symptoms. Broader symptom resolution likely requires longer or more intensive interventions integrated with workplace environmental controls.

### 4.4 Implementation implications and the occupational health nursing role

From an implementation perspective, on-site occupational health nurses delivered the program using a structured workflow embedded within routine workplace health services. Rather than serving solely as outcome assessors or health-surveillance providers, the nursing staff were responsible for the full operational chain—needs assessment, theory-based session delivery, between-session support, fidelity recording, and outcome data collection. The program therefore illustrates one way in which occupational health nurses may translate behavioral theory into routine workplace health-promotion activities, linking individual behavior change with organizational safety systems. The peer-supported ‘Workplace Health Guardian' structure, coordinated by the nursing staff, offers a candidate, potentially replicable model for embedding behavior-change support into existing occupational health-service delivery without requiring external personnel.

Self-reported preventive behavior at 82.4% 4 weeks after the active intervention period may reflect continued self-reported practice; the single-item dichotomous self-report measure cannot, however, distinguish actual behavioral occurrence from social-desirability-biased reporting, nor can it capture quality, frequency, duration, or sustained adherence. Programs of this kind are likely to be most useful when integrated with multi-level organizational strategies including ergonomic environmental redesign, supervisor engagement, and policy alignment (7, 15). The limited resource intensity required to deliver this intervention—four 60-min group sessions plus thrice-weekly short small-group activities, coordinated by existing occupational health nursing staff—suggests potential implementation feasibility in other manufacturing settings, subject to confirmatory evidence from adequately powered studies.

### 4.5 Limitations

Several limitations warrant emphasis. First, the two-cluster allocation precludes definitive separation of intervention effects from plant-level confounders; conventional cluster-randomized-trial inference standards cannot be met with only two clusters (21), and the findings are presented as preliminary program evaluation evidence rather than as definitive effectiveness data. Second, the study was not prospectively registered, representing a transparency limitation. Third, although baseline differences did not reach statistical significance for most characteristics, *p* > 0.05 with *n* = 69 cannot be interpreted as evidence of equivalence; in particular, the higher baseline NIOSH symptom-carrier rate in the intervention group (73.5 vs. 57.1%) and the lower baseline attitude score in the intervention group raise the possibility that residual imbalance and regression to the mean—whereby a more-symptomatic group naturally trends toward less symptomatic states over time—may partly account for the observed patterns. Fourth, health behavior practice was assessed using a single dichotomous self-report item that is susceptible to social-desirability bias and demand characteristics, and cannot capture frequency, duration, or quality of practiced behavior; the apparent magnitude of self-reported behavioral change should not be interpreted as objectively verified adherence. Fifth, the 4-week post-intervention follow-up limits inference about long-term behavioral maintenance and clinical sustainability. Sixth, the all-male single-industry sample from two plants in a single Korean city limits generalizability to female workers, other industries, and other regions. Seventh, although the plants were geographically and administratively separated, complete absence of cross-cluster contamination cannot be fully verified. Eighth, the study was not powered to detect clinical symptom outcomes; the body-region-specific symptom analyses were uncorrected for multiple testing (six body regions × three symptom subdomains × three timepoints) and some observed *p*-values below 0.05 may therefore reflect chance findings. Ninth, and importantly, the control group did not receive an attention-matched program. The intervention group received substantially greater contact time, structured peer engagement, and group support than controls, so a meaningful part of the observed changes in PBC, behavioral intention, and self-reported behavior may be attributable to non-specific attention, expectancy, and social-facilitation effects rather than to the TPB-specific components of the program. The present design cannot disentangle these mechanisms, and the behavioral findings should be read with this confound firmly in mind. Tenth, detailed individual-level ergonomic exposure variables (specific job task, repetitive-motion frequency, manual material handling load) were not collected, limiting adjustment for baseline biomechanical risk. Eleventh, the overall attrition rate of 9.32% over a short intervention period, although modest, suggests that participation-maintenance strategies would be useful for future scale-up. Twelfth, outcome assessors were not blinded to allocation; in combination with self-reported outcomes, this raises the possibility of reporting bias that available-case analysis cannot fully address.

### 4.6 Future research

Confirmatory research should comprise prospectively registered multi-site cluster randomized trials with adequate cluster numbers, longer follow-up of at least 3–6 months, objective measures of behavioral adherence (e.g., wearable accelerometry, supervisor-rated observation), inclusion of ergonomic and environmental outcomes, attention-matched control conditions to disentangle TPB-specific effects from non-specific attention effects, female and multi-industry samples, and economic evaluation. Mechanistic mediation analyses examining whether changes in PBC mediate downstream symptom change would further strengthen the evidence base. Integration of behavioral intervention components with engineering and organizational controls—the ‘person–environment–behavior' approach increasingly advocated in occupational and public health research (7, 15)—remains an important priority.

## 5 Conclusion

In this preliminary two-cluster workplace program evaluation, implementation of a TPB-based musculoskeletal health program—coordinated and delivered by on-site occupational health nursing staff—was accompanied by short-term changes at the individual level in perceived behavioral control, behavioral intention, and self-reported preventive health behavior among automobile manufacturing workers. Overall NIOSH musculoskeletal symptom-carrier status did not differ significantly between groups at any timepoint; exploratory body-region-specific analyses (Supplementary File S2) were uncorrected for multiplicity, inconsistent across subdomains and timepoints, and do not establish symptom benefit. Given the two-cluster design, non-attention-matched control condition, single-item self-reported behavioral outcome, possibility of residual baseline imbalance and regression to the mean, and absence of prospective registration, these findings should be interpreted as feasibility- and mechanism-oriented signals rather than as evidence of intervention efficacy. The program nonetheless illustrates one way in which occupational health nurses may embed behavioral theory within routine workplace health-promotion services—a candidate implementation model that warrants confirmatory evaluation in larger, prospectively registered, multi-site studies with adequate cluster numbers, attention-matched control conditions, and objective behavioral and symptom outcomes.

## Data Availability

The datasets presented in this article are not readily available because the dataset contains de-identified individual-level data collected from workers in two specific manufacturing plants. Although direct identifiers have been removed, the data include occupational, health-related, and workplace-specific information that may carry a risk of re-identification because of the small sample size and clustered workplace setting. Therefore, the dataset is not publicly available. De-identified data may be made available from the corresponding author upon reasonable request, subject to institutional approval, data-sharing policies, and protection of participant confidentiality. Requests to access the datasets should be directed to gaeunkim0325@gmail.com.
