# Supplementary material for: Short-term changes in behavioral determinants following a theory-based workplace musculoskeletal health program among automobile manufacturing workers: a two-cluster program evaluation
Source: Front Public Health. 2026 Jul 7;14:1888259. doi: 10.3389/fpubh.2026.1888259 (PMC13384917; doi:10.3389/fpubh.2026.1888259)
Supplement: Supplementary file 1 [file Table_1.docx]

**Supplementary File S1. CONSORT 2010 extension to cluster randomised trials — reporting checklist for the present two-cluster program evaluation**

*Manuscript: 'Short-Term Changes in Behavioral Determinants Following a Theory-Based Workplace Musculoskeletal Health Program among Automobile Manufacturing Workers: A Two-Cluster Program Evaluation'*

*This checklist follows the CONSORT 2010 extension for cluster randomised trials (Campbell MK, Piaggio G, Elbourne DR, Altman DG. Consort 2010 statement: extension to cluster randomised trials. BMJ. 2012;345:e5661. doi:10.1136/bmj.e5661). Because the present study was a two-cluster, cluster-allocated controlled workplace program evaluation rather than a fully powered multi-cluster randomised trial, the checklist is provided to transparently document reporting decisions; items not applicable or not met are stated as such. Section/page numbers refer to the main manuscript.*

| Section / item | Standard CONSORT item | Cluster extension | Reported on (manuscript location) |
| --- | --- | --- | --- |
| 1a — Title | Identification as a randomised trial in the title | Identification as a cluster randomised trial | Title identifies the study as 'A Two-Cluster Program Evaluation', explicitly avoiding 'trial' to reflect the preliminary, non-prospectively-registered design. |
| 1b — Abstract | Structured summary of trial design, methods, results, and conclusions | Structured summary including cluster design | Abstract is structured (Background/Methods/Results/Conclusion/Clinical Trial Registration). Cluster nature explicit in Methods sentence: 'A two-cluster, cluster-allocated controlled workplace program evaluation...'. |
| 2a — Background and objectives | Scientific background and explanation of rationale | Rationale for using a cluster design | Introduction §§ 1–5 (pages 2–3). Plant-level allocation rationale in Methods 2.1: 'Plant-level allocation was chosen to prevent cross-group contamination'. |
| 2b — Specific objectives | Specific objectives or hypotheses | Whether objectives pertain to the cluster level, individual participant level, or both | Introduction §5 (page 3): objectives stated at the individual worker level (behavioral determinants, behavior practice, musculoskeletal symptoms). |
| 3a — Trial design | Description of trial design including allocation ratio | Definition of cluster and description of how the design features apply to the clusters | Methods 2.1 (page 3): two-cluster cluster-allocated controlled program evaluation; cluster = manufacturing plant; allocation 1:1; explicit acknowledgement that this is not a fully powered multi-cluster RCT. |
| 3b — Important changes to methods | Important changes to methods after trial commencement | Reasons for changes | No changes made to methods after enrolment commenced (Methods 2.4, TIDieR item 10). |
| 4a — Eligibility | Eligibility criteria for participants | Eligibility criteria for clusters | Methods 2.2 (page 3): cluster eligibility = automobile manufacturing plants of comparable size (~200 workers each) in City D, with similar assembly-line processes. Individual-level eligibility (employment ≥1 year; musculoskeletal symptoms in past 12 months; exclusions for severe trauma, surgery, comorbidities). |
| 4b — Settings and locations | Settings and locations where the data were collected | — | Methods 2.2 (page 3): two manufacturing plants in City D, Republic of Korea. |
| 5 — Interventions | Interventions for each group with sufficient detail to allow replication, including how and when administered | Whether interventions pertain to the cluster level, individual participant level, or both | Methods 2.4 (pages 3–4); Table 2 (TIDieR); Table 3 (session-by-session). Intervention delivered at the individual participant level within the intervention-allocated cluster; control condition described at cluster level. |
| 6a — Outcomes | Completely defined pre-specified primary and secondary outcome measures, including how and when assessed | Whether outcome measures pertain to the cluster level, individual participant level, or both | Methods 2.5. All outcomes assessed at individual participant level. Outcomes of interest: PBC and behavioral intention. Additional outcomes: attitude, subjective norms, self-reported health behavior practice, and NIOSH-based musculoskeletal symptoms. Manuscript explicitly states that because the study was not prospectively registered, the primary/additional distinction should be regarded as analytic structure rather than a registered hypothesis hierarchy. |
| 6b — Changes to outcomes | Any changes to trial outcomes after the trial commenced, with reasons | — | No changes to outcomes after enrolment commenced. |
| 7a — Sample size | How sample size was determined | Method of calculation, including number of clusters, cluster size, coefficient of intracluster correlation (ICC), and an indication of its uncertainty | Methods 2.2: individual-level sample size calculation via G*Power 3.1.7 (α = 0.05, d = 0.8, power = 0.80), 26 per group plus 30% attrition → 37 per cluster. NOT POWERED at the cluster level (only two clusters available). This is openly acknowledged as a study limitation (Section 4.5, limitations 1 and 8). |
| 7b — Interim analyses | When applicable, explanation of any interim analyses and stopping guidelines | — | No interim analyses or stopping rules; not applicable. |
| 8a — Sequence generation | Method used to generate the random allocation sequence | Whether randomisation accounted for clusters | Methods 2.2 (page 3): two plants allocated using an opaque-envelope procedure for pragmatic assignment. Manuscript explicitly states: 'with only two clusters, this should not be interpreted as achieving randomization-based balance'. |
| 8b — Type of randomisation | Type of randomisation; details of any restriction | Details of stratification or matching if used | No stratification, matching, or restricted randomisation; allocation was a single envelope draw at the cluster level. This limitation is noted in Methods 2.2 and Limitations 1 (Section 4.5). |
| 9 — Allocation concealment | Mechanism used to implement the random allocation sequence | Specification of whether allocation concealment was at the cluster level, individual participant level, or both | Allocation at the cluster level via opaque envelope opened in the presence of plant representatives. Individual-level allocation concealment is not applicable because allocation was determined by cluster membership. |
| 10 — Implementation | Who generated the allocation sequence, who enrolled participants, and who assigned participants to interventions | Replace by 10a, 10b, and 10c. | Methods 2.2 (page 3). 10a: allocation sequence generated by the research team. 10b: occupational health nursing staff at each plant recruited individual workers within their allocated cluster. 10c: allocation concealment at the cluster (plant) level; individual-level allocation concealment not applicable. |
| 11a — Blinding | If done, who was blinded after assignment and how | Whether or not blinding was performed at the cluster level, individual participant level, or both | Methods 2.2 (page 3): workers were aware of their cluster's allocation; outcome assessors were not blinded. This is standard for pragmatic cluster-allocated workplace evaluations and is acknowledged. |
| 11b — Similarity of interventions | If relevant, description of the similarity of interventions | — | The intervention and control conditions are not designed to be similar; the control condition is plant-standard mandatory safety education. The attention asymmetry is explicitly acknowledged in Methods 2.4 and as Limitation 10 (Section 4.5). |
| 12a — Statistical methods | Statistical methods used to compare groups for primary and secondary outcomes | How clustering was taken into account | Methods 2.7. GEE with exchangeable working correlation structure was applied to model G/T/G × T effects for the four continuous TPB constructs at the individual level; dichotomous outcomes (behavior practice, NIOSH symptom carrier, body-region categories) were compared with Pearson chi-square at each timepoint. Manuscript explicitly states that GEE was used to describe individual-level repeated-measure trajectories transparently and not to overcome the inferential limitations of the two-cluster design. |
| 12b — Additional analyses | Methods for additional analyses, such as subgroup analyses and adjusted analyses | — | Methods 2.7: an additional exploratory analysis within the subgroup of workers with any symptom improvement (n = 32) is reported in Supplementary File S2. Body-region-specific symptom analyses are flagged as uncorrected exploratory throughout the manuscript. |
| 13a — Participant flow | For each group, numbers of participants randomly assigned, receiving intended treatment, and analysed for primary outcome | For each group, numbers of clusters that were randomly assigned, received intended treatment, and were analysed for the primary outcome | Figure 1 (CONSORT cluster flow diagram). Two clusters allocated; both received their intended condition; both contributed individual-level outcome data. Individual-level numbers: 74 enrolled → 69 analysed (intervention 34; control 35). |
| 13b — Losses and exclusions | For each group, losses and exclusions after randomisation, together with reasons | For each group, losses and exclusions for both clusters and individual participants | Cluster-level losses: none (both clusters retained). Individual-level losses: 5/74 withdrawals (9.32%) during the intervention period (intervention group 3; control group 2; reasons: health-related absence, business travel, resignation). Reasons reported in Methods 2.2. |
| 14a — Recruitment | Dates defining the periods of recruitment and follow-up | — | Methods 2.2 and 2.6: data collection between February and March 2025; follow-up 8 weeks from baseline. |
| 14b — Stopped early | Why the trial ended or was stopped | — | Study completed as planned; not applicable. |
| 15 — Baseline data | A table showing baseline demographic and clinical characteristics for each group | Baseline characteristics for the individual and cluster levels as applicable for each group | Table 1. Individual-level baseline characteristics for both groups. Cluster-level descriptors (plant size, assembly-line processes, administrative separation, geographic distance) provided narratively in Methods 2.1 and 2.2. |
| 16 — Numbers analysed | For each group, number of participants (denominator) included in each analysis and whether the analysis was by original assigned groups | For each group, number of clusters included in each analysis | Tables 1, 4, 5, 6. Both clusters included in every analysis; individual-level n = 34 and 35 in intervention and control, respectively. Available-case analysis (missing-at-random assumption). |
| 17a — Outcomes and estimation | For each primary and secondary outcome, results for each group, and the estimated effect size and its precision | Results at the individual or cluster level as applicable and a coefficient of intracluster correlation (ICC) for each primary outcome | Tables 4, 5, 6 (individual-level outcomes with descriptive statistics, GEE main and interaction effects, and chi-square between-group comparisons for dichotomous outcomes). |
| 17b — Binary outcomes | For binary outcomes, presentation of both absolute and relative effect sizes is recommended | — | Table 5: absolute proportions per timepoint and chi-square comparisons. Table 6: absolute proportions for NIOSH symptom-carrier status and body-region-specific symptom categories. |
| 18 — Ancillary analyses | Results of any other analyses performed, including subgroup analyses and adjusted analyses, distinguishing pre-specified from exploratory | — | Section 3.5 (brief reference) and Supplementary File S2 (full results). Two exploratory analyses are reported: (a) TPB construct changes within the symptom-improvement subgroup (n = 32), explicitly labeled as outcome-defined and vulnerable to selection bias; (b) full body-region-specific symptom data across six regions × three subdomains × three timepoints, with multiplicity caveats applied throughout. Both are framed as hypothesis-generating only. |
| 19 — Harms | All important harms or unintended effects in each group | — | No adverse events or unintended effects observed or reported by participants during the 4-week intervention or 4-week follow-up period. |
| 20 — Limitations | Trial limitations, addressing sources of potential bias, imprecision, and, if relevant, multiplicity of analyses | — | Discussion 4.5, 12 numbered limitations: two-cluster design (1), non-registration (2), residual baseline imbalance and regression to the mean (3), single-item self-reported behavior with social-desirability and demand-characteristic risk (4), short follow-up (5), all-male single-industry sample (6), residual contamination risk (7), uncorrected multiplicity in body-region-specific symptom analyses (8), non-attention-matched control (9), missing ergonomic exposure variables (10), 9.32% attrition (11), unblinded outcome assessment with self-reported outcomes (12). |
| 21 — Generalisability | Generalisability (external validity, applicability) of the trial findings | Generalisability to clusters and/or individual participants (as relevant) | Discussion 4.3 (public health implications) and 4.4 (implementation implications). Generalisability acknowledged as limited because of single-industry, all-male sample and two-cluster design. |
| 22 — Interpretation | Interpretation consistent with results, balancing benefits and harms, and considering other relevant evidence | — | Discussion 4.1–4.6 (pages 6–8). Interpretation framed as preliminary, hypothesis-generating evidence rather than as definitive effectiveness. |
| 23 — Registration | Registration number and name of trial registry | — | The study was not prospectively or retrospectively registered. This is openly reported as a transparency limitation in the Abstract (Clinical Trial Registration), Methods 2.3, Limitations 2 (Section 4.5), and Ethics Statement. |
| 24 — Protocol | Where the full trial protocol can be accessed, if available | — | No publicly accessible protocol; the study was implemented as an institutionally approved workplace health promotion program evaluation. IRB approval (No. 40525-202410-HR-062-03) documented in Methods 2.3 and Ethics Statement. |
| 25 — Funding | Sources of funding and other support; role of funders | — | — |
