# Supplementary material for: Short-term changes in behavioral determinants following a theory-based workplace musculoskeletal health program among automobile manufacturing workers: a two-cluster program evaluation
Source: Front Public Health. 2026 Jul 7;14:1888259. doi: 10.3389/fpubh.2026.1888259 (PMC13384917; doi:10.3389/fpubh.2026.1888259)
Supplement: Supplementary file 2 [file Table_2.docx]

**Supplementary File S2. Exploratory analyses**

*Manuscript: 'Short-Term Changes in Behavioral Determinants Following a Theory-Based Workplace Musculoskeletal Health Program among Automobile Manufacturing Workers: A Two-Cluster Program Evaluation'*

**Part 1. Symptom-improvement subgroup analysis (n = 32)**

Among the 69 analyzed workers, 32 (intervention group n = 17; control group n = 15) showed any improvement in self-reported musculoskeletal symptoms—operationalized as a positive change in at least one of duration, intensity, or frequency in any body region between baseline and any subsequent timepoint. Within this post-hoc-defined subgroup, between-group differences in TPB construct scores were examined at each timepoint using independent-samples t-tests.

**Supplementary Table S2-1. TPB construct scores in the symptom-improvement subgroup (n = 32).**

| Construct / Time | Intervention (n = 17), M ± SD | Control (n = 15), M ± SD | t | p |
| --- | --- | --- | --- | --- |
| Attitude |  |  |  |  |
| Baseline | 3.00 ± 0.77 | 3.33 ± 0.79 | −1.21 | 0.237 |
| Post | 3.54 ± 1.20 | 3.48 ± 0.73 | 0.18 | 0.863 |
| Follow-up | 3.09 ± 0.85 | 3.48 ± 0.73 | −1.40 | 0.172 |
| Subjective norms |  |  |  |  |
| Baseline | 3.22 ± 0.77 | 2.85 ± 0.81 | 1.33 | 0.194 |
| Post | 3.47 ± 1.13 | 2.87 ± 0.87 | 1.68 | 0.103 |
| Follow-up | 3.32 ± 0.99 | 2.82 ± 0.82 | 1.57 | 0.128 |
| Perceived behavioral control |  |  |  |  |
| Baseline | 3.03 ± 0.68 | 3.07 ± 1.19 | −0.11 | 0.916 |
| Post | 3.43 ± 0.92 | 3.08 ± 1.31 | 0.85 | 0.404 |
| Follow-up | 3.26 ± 0.76 | 2.92 ± 1.19 | 0.97 | 0.342 |
| Behavioral intention |  |  |  |  |
| Baseline | 3.13 ± 1.00 | 3.35 ± 0.47 | −0.80 | 0.431 |
| Post | 3.87 ± 1.12 | 3.20 ± 0.59 | 2.14 | 0.042* |
| Follow-up | 3.87 ± 1.12 | 3.15 ± 0.52 | 2.36 | 0.027* |

*M, mean; SD, standard deviation. *p < 0.05, uncorrected. The subgroup was defined by post-baseline symptom improvement at any timepoint and is therefore not randomly defined. Reported p-values are descriptive and are not adjusted for multiple comparisons across constructs, timepoints, or the parent body-region symptom analyses from which the subgroup was derived. Reproduced from the underlying doctoral dissertation (Table 5).*

Interpretation: within the symptom-improvement subgroup, intervention-group workers reported higher behavioral intention than control-group workers at post-intervention and follow-up. No statistically significant between-group differences were observed for attitude, subjective norms, or PBC at any timepoint within this subgroup. Because the subgroup is outcome-defined, this pattern cannot be interpreted as evidence that symptom improvement is mediated by TPB change, nor as evidence that TPB change caused symptom improvement; both could be co-occurring consequences of engagement, peer effects, or non-specific attention.

**Part 2. Full body-region-specific musculoskeletal symptom data**

The main-text Table 6 summarizes only those body-region-specific symptom subdomains that reached an uncorrected between-group p < 0.05. The full body-region-specific data are provided here in three tables (S2-2 to S2-4): symptom duration, intensity, and frequency for each of six body regions (neck, shoulder, arm, hand, back, leg) at three timepoints (baseline, post-intervention, follow-up). Data are restricted to participants who reported a symptom in the respective region at the corresponding timepoint, so denominators vary across regions and timepoints.

**Supplementary Table S2-2. Body-region-specific symptom data: neck and shoulder.**

| Region / Subdomain / Category | I baseline n(%) | C baseline n(%) | p (baseline) | | I post n(%) | C post n(%) | p (post) | I follow-up n(%) | C follow-up n(%) | p (follow-up) |
| --- | --- | --- | --- | --- | --- | --- | --- | --- | --- | --- |
| NECK — Duration |  |  | 0.308 |  | |  | 0.104 |  |  | 0.104 |
| < 1 day | 7 (43.8) | 2 (22.2) |  | 8 (50.0) | | 1 (12.5) |  | 8 (50.0) | 1 (12.5) |  |
| 1 day – < 1 week | 4 (25.0) | 1 (11.1) |  | 6 (37.5) | | 3 (37.5) |  | 6 (37.5) | 3 (37.5) |  |
| ≥ 1 week | 5 (31.3) | 6 (66.7) |  | 2 (12.5) | | 4 (50.0) |  | 2 (12.5) | 4 (50.0) |  |
| NECK — Intensity |  |  | 0.012* |  | |  | 0.007* |  |  | 0.007* |
| Mild | 15 (93.8) | 4 (44.4) |  | 15 (93.8) | | 3 (37.5) |  | 15 (93.8) | 3 (37.5) |  |
| Moderate | 1 (6.3) | 4 (44.4) |  | 1 (6.3) | | 4 (50.0) |  | 1 (6.3) | 4 (50.0) |  |
| Severe | 0 (0.0) | 1 (11.1) |  | 0 (0.0) | | 1 (12.5) |  | 0 (0.0) | 1 (12.5) |  |
| NECK — Frequency |  |  | 0.813 |  | |  | 0.690 |  |  | 0.695 |
| Once / 6 months | 2 (12.5) | 2 (22.2) |  | 1 (6.7) | | 2 (22.2) |  | 1 (6.7) | 2 (22.2) |  |
| Once / 2–3 months | 2 (12.5) | 1 (11.1) |  | 4 (26.7) | | 2 (22.2) |  | 5 (33.3) | 2 (22.2) |  |
| ≥ Monthly | 12 (75.0) | 6 (66.7) |  | 10 (66.7) | | 5 (55.6) |  | 9 (60.0) | 5 (55.6) |  |
| SHOULDER — Duration |  |  | 0.587 |  | |  | 0.842 |  |  | 0.587 |
| < 1 day | 1 (6.3) | 1 (10.0) |  | 1 (6.3) | | 1 (10.0) |  | 1 (6.3) | 1 (10.0) |  |
| 1 day – < 1 week | 10 (62.5) | 4 (40.0) |  | 10 (62.5) | | 5 (50.0) |  | 10 (62.5) | 4 (40.0) |  |
| ≥ 1 week | 5 (31.3) | 5 (50.0) |  | 5 (31.3) | | 4 (40.0) |  | 5 (31.3) | 5 (50.0) |  |
| SHOULDER — Intensity |  |  | 0.029* |  | |  | 0.029* |  |  | 0.029* |
| Mild | 11 (68.8) | 3 (30.0) |  | 11 (68.8) | | 3 (30.0) |  | 11 (68.8) | 3 (30.0) |  |
| Moderate | 3 (18.8) | 7 (70.0) |  | 3 (18.8) | | 7 (70.0) |  | 3 (18.8) | 7 (70.0) |  |
| Severe | 2 (12.5) | 0 (0.0) |  | 2 (12.5) | | 0 (0.0) |  | 2 (12.5) | 0 (0.0) |  |
| SHOULDER — Frequency |  |  | 0.631 |  | |  | 0.063 |  |  | 0.183 |
| Once / 6 months | 0 (0.0) | 1 (10.0) |  | 0 (0.0) | | 1 (10.0) |  | 0 (0.0) | 1 (10.0) |  |
| Once / 2–3 months | 1 (6.3) | 0 (0.0) |  | 10 (62.5) | | 2 (20.0) |  | 6 (37.5) | 1 (10.0) |  |
| ≥ Monthly | 15 (93.8) | 9 (90.0) |  | 6 (37.5) | | 7 (70.0) |  | 10 (62.5) | 8 (80.0) |  |

**p < 0.05, uncorrected. I, intervention group; C, control group. Denominators are participants who reported a neck or shoulder symptom at the respective timepoint. Reproduced from underlying doctoral dissertation Appendix 10-A.*

**Supplementary Table S2-3. Body-region-specific symptom data: arm and hand.**

| Region / Subdomain / Category | I baseline n(%) | C baseline n(%) | p (baseline) | I post n(%) | C post n(%) | p (post) | I follow-up n(%) | C follow-up n(%) | p (follow-up) |
| --- | --- | --- | --- | --- | --- | --- | --- | --- | --- |
| ARM — Duration |  |  | >0.999 |  |  | >0.999 |  |  | 0.580 |
| < 1 day | 5 (62.5) | 4 (57.1) |  | 5 (62.5) | 4 (57.1) |  | 5 (62.5) | 4 (66.7) |  |
| 1 day – < 1 week | 1 (12.5) | 2 (28.6) |  | 1 (12.5) | 2 (28.6) |  | 1 (12.5) | 2 (33.3) |  |
| ≥ 1 week | 2 (25.0) | 1 (14.3) |  | 2 (25.0) | 1 (14.3) |  | 2 (25.0) | 0 (0.0) |  |
| ARM — Intensity |  |  | 0.119 |  |  | 0.119 |  |  | 0.138 |
| Mild | 3 (37.5) | 6 (85.7) |  | 3 (37.5) | 6 (85.7) |  | 3 (37.5) | 5 (83.3) |  |
| Moderate | 5 (62.5) | 1 (14.3) |  | 5 (62.5) | 1 (14.3) |  | 5 (62.5) | 1 (16.7) |  |
| Severe | 0 (0.0) | 0 (0.0) |  | 0 (0.0) | 0 (0.0) |  | 0 (0.0) | 0 (0.0) |  |
| ARM — Frequency |  |  | 0.013* |  |  | 0.002* |  |  | 0.041* |
| Once / 6 months | 1 (12.5) | 2 (28.6) |  | 2 (25.0) | 2 (28.6) |  | 2 (25.0) | 2 (33.3) |  |
| Once / 2–3 months | 1 (12.5) | 5 (71.4) |  | 0 (0.0) | 5 (71.4) |  | 1 (12.5) | 4 (66.7) |  |
| ≥ Monthly | 6 (75.0) | 0 (0.0) |  | 6 (75.0) | 0 (0.0) |  | 5 (62.5) | 0 (0.0) |  |
| HAND — Duration |  |  | 0.515 |  |  | 0.436 |  |  | 0.663 |
| < 1 day | 3 (23.1) | 7 (36.8) |  | 2 (16.7) | 7 (36.8) |  | 3 (23.1) | 5 (29.4) |  |
| 1 day – < 1 week | 9 (69.2) | 9 (47.4) |  | 9 (75.0) | 9 (47.4) |  | 9 (69.2) | 9 (52.9) |  |
| ≥ 1 week | 1 (7.7) | 3 (15.8) |  | 1 (8.3) | 3 (15.8) |  | 1 (7.7) | 3 (17.6) |  |
| HAND — Intensity |  |  | 0.142 |  |  | 0.452 |  |  | 0.112 |
| Mild | 4 (30.8) | 8 (42.1) |  | 6 (50.0) | 6 (31.6) |  | 4 (30.8) | 9 (52.9) |  |
| Moderate | 6 (46.2) | 11 (57.9) |  | 6 (50.0) | 13 (68.4) |  | 6 (46.2) | 8 (47.1) |  |
| Severe | 3 (23.1) | 0 (0.0) |  | 0 (0.0) | 0 (0.0) |  | 3 (23.1) | 0 (0.0) |  |
| HAND — Frequency |  |  | 0.320 |  |  | 0.254 |  |  | 0.206 |
| Once / 6 months | 6 (50.0) | 6 (33.3) |  | 7 (58.3) | 5 (27.8) |  | 5 (41.7) | 7 (41.2) |  |
| Once / 2–3 months | 5 (41.7) | 6 (33.3) |  | 5 (41.7) | 11 (61.1) |  | 7 (58.3) | 6 (35.3) |  |
| ≥ Monthly | 1 (8.3) | 6 (33.3) |  | 0 (0.0) | 2 (11.1) |  | 0 (0.0) | 4 (23.5) |  |

**p < 0.05, uncorrected. I, intervention group; C, control group. Denominators are participants who reported an arm or hand symptom at the respective timepoint. Reproduced from underlying doctoral dissertation Appendix 10-B.*

**Supplementary Table S2-4. Body-region-specific symptom data: back and leg.**

| Region / Subdomain / Category | I baseline n(%) | C baseline n(%) | p (baseline) | I post n(%) | C post n(%) | p (post) | I follow-up n(%) | C follow-up n(%) | p (follow-up) |
| --- | --- | --- | --- | --- | --- | --- | --- | --- | --- |
| BACK — Duration |  |  | 0.790 |  |  | 0.776 |  |  | 0.371 |
| < 1 day | 4 (44.4) | 3 (60.0) |  | 4 (44.4) | 4 (66.7) |  | 4 (44.4) | 3 (60.0) |  |
| 1 day – < 1 week | 4 (44.4) | 1 (20.0) |  | 4 (44.4) | 1 (16.7) |  | 5 (55.6) | 1 (20.0) |  |
| ≥ 1 week | 1 (11.1) | 1 (20.0) |  | 1 (11.1) | 1 (16.7) |  | 0 (0.0) | 1 (20.0) |  |
| BACK — Intensity |  |  | >0.999 |  |  | >0.999 |  |  | 0.545 |
| Mild | 6 (66.7) | 3 (50.0) |  | 4 (44.4) | 2 (33.3) |  | 6 (66.7) | 2 (40.0) |  |
| Moderate | 2 (22.2) | 2 (33.3) |  | 4 (44.4) | 3 (50.0) |  | 2 (22.2) | 3 (60.0) |  |
| Severe | 1 (11.1) | 1 (16.7) |  | 1 (11.1) | 1 (16.7) |  | 1 (11.1) | 0 (0.0) |  |
| BACK — Frequency |  |  | 0.329 |  |  | 0.545 |  |  | 0.248 |
| Once / 6 months | 2 (22.2) | 2 (40.0) |  | 2 (22.2) | 3 (60.0) |  | 2 (22.2) | 3 (60.0) |  |
| Once / 2–3 months | 0 (0.0) | 1 (20.0) |  | 1 (11.1) | 0 (0.0) |  | 1 (11.1) | 1 (20.0) |  |
| ≥ Monthly | 7 (77.8) | 2 (40.0) |  | 6 (66.7) | 2 (40.0) |  | 6 (66.7) | 1 (20.0) |  |
| LEG — Duration |  |  | 0.184 |  |  | 0.158 |  |  | 0.152 |
| < 1 day | 3 (37.5) | 2 (20.0) |  | 3 (37.5) | 3 (27.3) |  | 2 (25.0) | 3 (30.0) |  |
| 1 day – < 1 week | 2 (25.0) | 7 (70.0) |  | 3 (37.5) | 8 (72.7) |  | 3 (37.5) | 7 (70.0) |  |
| ≥ 1 week | 3 (37.5) | 1 (10.0) |  | 2 (25.0) | 0 (0.0) |  | 3 (37.5) | 0 (0.0) |  |
| LEG — Intensity |  |  | 0.079 |  |  | 0.607 |  |  | 0.079 |
| Mild | 5 (62.5) | 7 (70.0) |  | 5 (62.5) | 7 (63.6) |  | 5 (62.5) | 7 (70.0) |  |
| Moderate | 0 (0.0) | 3 (30.0) |  | 2 (25.0) | 4 (36.4) |  | 0 (0.0) | 3 (30.0) |  |
| Severe | 3 (37.5) | 0 (0.0) |  | 1 (12.5) | 0 (0.0) |  | 3 (37.5) | 0 (0.0) |  |
| LEG — Frequency |  |  | 0.075 |  |  | 0.811 |  |  | 0.067 |
| Once / 6 months | 1 (12.5) | 5 (50.0) |  | 2 (28.6) | 5 (50.0) |  | 1 (14.3) | 5 (50.0) |  |
| Once / 2–3 months | 0 (0.0) | 2 (20.0) |  | 1 (14.3) | 2 (20.0) |  | 0 (0.0) | 2 (20.0) |  |
| ≥ Monthly | 7 (87.5) | 3 (30.0) |  | 4 (57.1) | 3 (30.0) |  | 6 (85.7) | 3 (30.0) |  |

**p < 0.05, uncorrected. I, intervention group; C, control group. Denominators are participants who reported a back or leg symptom at the respective timepoint. Reproduced from underlying doctoral dissertation Appendix 10-C.*
